# Transcranial Magnetic Stimulation for the Treatment of Chemo Brain

**DOI:** 10.3390/s23198017

**Published:** 2023-09-22

**Authors:** Phillip H. Kuo, Allison Yu-Chin Chen, Rudolph J. Rodriguez, Carol Stuehm, Pavani Chalasani, Nan-Kuei Chen, Ying-Hui Chou

**Affiliations:** 1 Departments of Medical Imaging, Medicine, and Biomedical Engineering, University of Arizona, Tucson, AZ 85721, USA; 2 Brain Imaging and TMS Laboratory, Department of Psychology, University of Arizona, Tucson, AZ 85721, USA; yuchinchen@arizona.edu; 3 Department of Physics, University of Colorado, Boulder, CO 80301, USA; rudolph.rodriguez@colorado.edu; 4 Department of Medical Imaging, University of Arizona, Tucson, AZ 85721, USA; carols@arizona.edu; 5 Division of Hematology-Oncology, George Washington Cancer Center, Washington, DC 20037, USA; pavani.chalasani@gwu.edu; 6 Department of Biomedical Engineering, University of Arizona, Tucson, AZ 85721, USA; nkchen@arizona.edu; 7 Brain Imaging and TMS Laboratory, Department of Psychology, Evelyn F McKnight Brain Institute, Arizona Center on Aging, BIO5 Institute, University of Arizona, Tucson, AZ 85721, USA; yinghuichou@email.arizona.edu

**Keywords:** transcranial magnetic stimulation, chemo brain, chemotherapy-related cognitive impairment

## Abstract

This pilot feasibility study aimed to evaluate the effects of transcranial magnetic stimulation (TMS) on chemotherapy-related cognitive impairment (CRCI), and we report here on the first patient. Background: Deleterious cognitive changes due to chemotherapy or CRCI are commonly referred to as “chemo brain”. With the increasing survival of cancer patients, this poorly understood and inadequately treated condition will likewise have an increasing toll on individuals and society. Since there is no approved treatment for chemo brain, we have initiated a therapeutic trial using transcranial magnetic stimulation (TMS), a non-invasive brain stimulation technique approved in many countries for the treatment of neurologic and psychiatric conditions like migraine and depression. Case presentation: A 58-year-old woman, diagnosed 7 years prior with left breast cancer, underwent partial mastectomy with sentinel lymph node biopsy. She then received four cycles of adjuvant chemotherapy followed by radiation therapy. Afterwards, she was on tamoxifen for 4 years and then switched to aromatase inhibitors. The patient’s CRCI started during chemotherapy and severely impaired her quality of life for an additional two years. In the third year after chemotherapy, the CRCI partially cleared to stabilize to the level at the time of presentation for this trial. The patient continues to have memory difficulties and decreased concentration, which makes multi-tasking very difficult to impossible. She is reliant on memory aids at work and at home. The participant underwent 10 consecutive sessions of TMS during weekdays for 2 weeks. Stimulation was directed to the left dorsolateral prefrontal cortex. After TMS, the participant significantly improved in memory function on neuropsychological testing. While she reported no subjective differences in concentration or memory, she did report an improvement in her sleep. Functional magnetic resonance imaging of the brain before and after TMS showed increased resting-state functional connectivity between the stimulation site and several brain regions. Remarkably, after 6 years of chemo brain and remaining in the same position at work due to her inability to concentrate and multi-task, she applied for and received a promotion 5–6 months after her TMS treatments. Conclusions: This first patient in the phase 1 clinical trial testing of TMS for the treatment of “chemo brain” provided important lessons for feasibility and insights into mechanisms of potential benefit.

## 1. Background

Chemo brain goes by many names, like chemotherapy-related cognitive impairment (CRCI), and is defined as cognitive changes that occur during and/or after chemotherapy. These changes may include the impairment of memory, learning, concentration, executive function, and visuo-spatial skills [1]. Additionally, anxiety and depression often co-exist with the cognitive symptoms and may be an inseparable component of the disease. The extent and duration of CRCI varies from mild and transient to severe and long-term, with significant detriment on quality of life [2,3,4,5,6,7,8]. The incidence of CRCI in breast cancer survivors varies widely, with some estimates suggesting it may affect up to 75% [9,10]. A review [9] indicated that the mean age of patients with CRCI in current studies was 50, and only six studies reported patients older than 50, with just two including women over 65, even though half of breast cancer patients are over 65. The total cost is not only direct, because of the consumption of resources for the treatment of symptoms, but also indirect due to loss of productivity. With more patients surviving longer with cancer or beating cancer entirely, CRCI will become an even more important issue in cancer survivorship, emerging as a heavy financial and social burden on society [11,12,13,14,15]. Currently, no treatment is approved for CRCI, and thus research into the understanding and treatment of CRCI needs to keep pace with our improving treatment of cancer.

There is still no clear understanding regarding the mechanisms involved in CRCI. Some theories suggest that cytotoxic antitumoral drugs inhibit enzymes that allow DNA replication or interfere with DNA synthesis and its components, leading to antiproliferative effects that can also negatively impact neurons throughout the body, resulting in neurotoxicity or reduced neurogenesis [16]. Beyond neurodegeneration, an animal model of CRCI showed that the complicated processes of neuronal signaling and long-term potentiation (LTP) might also be disrupted as a consequence of chemotherapy [17]. Although many antitumoral drugs may not be able to cross the blood–brain barrier (BBB), there are other possible indirect pathways that could affect the central nervous system. For instance, when mitochondrial DNA is damaged, oxidative stress and reactive oxygen species (ROS) increase because mitochondria lack DNA repair systems, leading to neuroinflammation and the cytokine-mediated disruption of the BBB [18]. In the neuroimaging modalities, magnetic resonance imaging (MRI) and positron emission tomography (PET) have been utilized to explore the pathophysiology of CRCI. Most of this research has been conducted in breast cancer patients and is the focus here. A study in breast cancer patients measured gray matter density (GMD) using an MRI and found decreases in GMD in the frontal, temporal, and cerebellar regions at one month post completion of chemotherapy, with a return to baseline levels in some but not all regions at one year after the completion of chemotherapy [19]. A prospective, longitudinal study utilizing functional MRI (fMRI) to assess cognitive task-related brain activation reported frontal hyper-activation prior to receiving systemic adjuvant therapy compared to healthy controls. After completing chemotherapy, patients did not maintain this hyper-activation, possibly due to the impairment of brain function by the chemotherapy [20]. PET perfusion imaging during the performance of memory-related tasks demonstrated altered blood flow in the frontal cortex and cerebellum [21].

The intricate situation draws parallels with treatment-resistant depression (TRD), where transcranial magnetic stimulation (TMS) has been FDA-approved to efficaciously alleviate symptoms of TRD. TMS is a non-invasive and safe magnetic brain stimulation technique which does not involve any form of general anesthesia or sedation. Since its introduction by Barker et al. in 1985 [22], more than 20,000 articles on TMS have been published. This neurostimulation technique is based on Faraday’s principle of electromagnetic induction. More specifically, the TMS coil creates a secondary electrical current that modulates the excitability of the underlying neurons and, thus, neural activity within specific regions of the brain [23]—commonly referred to as neuroplasticity. Neuroplasticity encompasses the ramifications of neuronal reorganization at the molecular, synaptic, and morphometric strata, thereby enabling the brain to undergo transformations from the dysfunctional state [24]. Neuroplasticity may also be characterized by the improvement of brain functionality arising from changes in neural circuits due to external stimuli, TMS being a notable example. TMS utilizes numerous different protocols. The repetitive TMS (rTMS) paradigm is one of the most used TMS protocols. It utilizes trains of pulses to induce cortical effects that outlast the duration of stimulation. rTMS allows researchers and clinicians to induce long-lasting changes in cortical reactivity and plasticity—long-term potentiation and depression (LTP/LTD)-like effects [24,25]. These mechanisms exhibit great potential for effectively alleviating cognitive deficits arising from CRCI. In clinical practice, a number of TMS devices and protocols have been approved by the United States Food and Drug Administration (USFDA) for the treatment of medication-refractory depression, migraine, obsessive compulsive disorder, and smoking addiction. Other approvals include pre-surgical motor and language mapping. Currently, TMS has been under investigation as a treatment tool in diverse disease states including Parkinson’s disease, Alzheimer’s disease, schizophrenia, stroke, epilepsy, autism, and tinnitus [26,27,28,29]. Although rTMS has been widely used in other neurological and psychiatric disorders and utilized to target cognitive impairments such as working memory, cognitive flexibility, and executive function, its efficacy has not yet been studied in patients with CRCI [30]. Given the unmet need to help patients with “chemo brain” and the proven ability of rTMS to treat neurologic conditions, we initiated a phase 1 clinical trial to assess the feasibility and potential efficacy of rTMS for the treatment of CRCI. 

In 2018, the USFDA approved an intermittent theta burst stimulation (iTBS) treatment, a form of shorter rTMS that facilitates cortical excitability, as a protocol stimulating the left dorsolateral prefrontal cortex (DLPFC) for the treatment of major depressive disorder. Cumulative evidence has indicated the effectiveness of excitatory iTBS over the left DLPFC in improving cognitive performance in psychiatric/neurological diseases or healthy volunteers [31,32]. To be precise, chemotherapy can adversely affect the structure of the brain by reducing the integrity of the white matter, such as through axon injuries and demyelination, primarily within the prefrontal cortex (PFC, where DLPFC is located) [10]. It is worth noting that the PFC, alongside its intricate network interlinking downstream cerebral structures, significantly contributes to the pathogenesis of depression. In light of these considerations, it appears that there may be a congruent repetitive TMS target, the left DLPFC, in TRD treatment that is potentially capable of addressing chemo brain’s challenges. For this study, the left DLPFC was therefore chosen as the iTBS target since it is a key element of many high-order brain functions, including the control of inhibition, attention, working memory, and decision-making, most of which happen to be functions significantly affected by CRCI. We used various tasks to detect changes in these cognitive functions, including Forward and Backward Digit Span for working memory, Rey Auditory Verbal Learning Test (RAVLT) for verbal working memory, verbal fluency task for semantic memory, and Stroop Task for cognitive flexibility and response inhibition. Moreover, the DLPFC is both structurally and functionally connected to many cortical and sub-cortical regions. Through either connectivity or network-wise interactions, stimulating the DLPFC could have effects on anatomically remote regions of the brain. To ensure the precise targeting of the DLPFC, sensors and advanced medical imaging are ingeniously combined. The exquisite structural anatomy of the brain provided by MRI is functionally mapped using sensors to ensure the high fidelity of the three-dimensional stereotactic navigation system and to measure the electromyography upon the targeted stimulation of serial brain regions. When scrutinized using the advanced neuroimaging technique, functional MRI (fMRI), the rTMS after-effects can be visibly revealed through changes in functional connectivity [24].

The primary aim is to determine the feasibility of iTBS for the treatment of CRCI. A secondary aim is to examine potential efficacy measured by standard neuropsychological testing and functional MRI. Our hypothesis is that excitatory iTBS exerts its effect by improving brain activity at the stimulation locus, thereby potentially eliciting neuroplasticity. This method distinctly focuses on the PFC, specifically DLPFC, the region most susceptible to CRCI, with the aim of counteracting the disruption of LTP and neural circuits. This strategic intervention holds the promise of enhancing intricate cerebral functions, consequently offering a prospective avenue for the amelioration of CRCI. 

## 2. Case Presentation

We obtained the consent of a 58-year-old right-handed white woman diagnosed 7 years prior with left breast cancer. She underwent partial mastectomy with sentinel lymph node biopsy and was found to have a 1 cm, grade III invasive ductal carcinoma with one out of four sentinel lymph nodes positive for cancer. The tumor was hormone receptor positive and human epidermal growth factor receptor 2 negative. Computed tomography and a bone scan were negative for distant metastatic disease. She then received four cycles of adjuvant chemotherapy with docetaxel and cyclophosphamide, followed by radiation therapy. Afterwards, she was started on adjuvant tamoxifen as she was premenopausal at diagnosis. She was on tamoxifen for 4 years, and then switched to aromatase inhibitors. Anastrozole was tried initially, but later a switch was made to letrozole, which continued to be the treatment at the time of her consenting to our study. Her only other medication was citalopram for sleep and stress, which she had been taking stably in the long term since before her treatment for breast cancer. 

The patient described how her “brain fog” started during chemotherapy and severely impaired her quality of life for an additional two years. She had no neuropathy or any other lasting side effects from her chemotherapy. In the third year after chemotherapy, her chemotherapy-related cognitive impairment partially cleared to stabilize to the level at the time of presentation for this study. The patient continues to have memory difficulties and decreased concentration, which makes multi-tasking very difficult to impossible. She is reliant on memory aids at work and has passed up opportunities for promotion, given her cognitive difficulties. At home, she is also reliant on memory aids and family members know that she is forgetful and ask her to write things down to remind herself.

## 3. Procedures and Data Analyses

This study was approved by the local Institutional Review Board, and informed written consent was obtained from the subject. The participant is a native English speaker with corrected to normal auditory and visual acuity. Neuropsychological exams, including the Uniform Data Set version 3 (UDS3) neuropsychological battery, were administered to measure cognitive function. The scores were normalized to z-scores for UDS3, with considerations for age, gender, and education, whereas the Rey Auditory Verbal Learning Test (RAVLT) scores were specifically adjusted for age with Mayo’s Older Americans Normative Studies (MOANS) [33,34,35]. Mild cognitive impairment was confirmed by standard criteria (i.e., any single neuropsychological measure 1.5 standard deviations lower than the normative mean regardless of the cognitive domains) [36]. In particular, her age-adjusted z-scores for Learning Efficiency Sum (LES) and Delay Recall Sum (DRS) on the RAVLT were −2.2 and −1.2, respectively. This indicates that her cognitive deficit was specific to verbal memory.

TMS parameters were established following an evaluation of safety, feasibility, and efficacy. The participant underwent 10 consecutive sessions of iTBS during weekdays over the course of two weeks, ensuring robust and reliable outcomes [26]. The iTBS protocol was employed with a MagVenture MagPro X100 stimulator (MagVenture Inc., Farum, Denmark) equipped with figure-of-eight magnetic coils (MagVenture C-B60 and Cool-B65 coils, MagVenture Inc., Farum, Denmark). iTBS pulses were delivered for a total of 600 pulses within a 192 s session, comprising repeated triplet bursts at 50 Hz for 2 s “on” (with 30 pulses) windows followed by 8 s “off” windows (with no pulses) [37]. The resting motor threshold (RMT) refers to the minimum stimulation intensity needed to produce at least 50 μV peak-to-peak amplitude of the right first dorsal interosseous muscle recorded by a sensor to capture the electromyography, and was determined by the parametric estimation via sequential testing (PEST) [38]. The initial planned 70% RMT was considered safe [39] and produced consistent facilitation [40] for this feasibility study. We implemented pre- and post-TMS session checklists to diligently assess any discomfort arising from the previous and the present TMS sessions, and we encouraged the participant to promptly communicate any discomfort or concerns during TMS intervention that they may have experienced. Earplugs were used to protect the participant’s hearing. A stereotactic 3D Neuronavigation system (LOCALITE, Bonn, Germany) was used to monitor and record the position and orientation of the stimulation coil in real time for consistency across iTBS sessions. The MNI coordinate of the stimulation site (i.e., left DLPFC) [−38, 44, 26] [41] was localized with the T1-MPRAGE anatomical MRI through the Neuronavigation system.

MRI sequences, T1-MPRAGE anatomical MRI and resting-state functional MRI (RS-fMRI), were acquired on a Siemens Skyra 3 Tesla MRI scanner (Siemens Medical Systems, Erlangen, Germany) with a 32-channel receiver head coil before and after the 10 iTBS sessions. Compared with task-based fMRI (tb-fMRI), we opted for rs-fMRI for several compelling reasons: (1) Participant Accessibility: tb-fMRI necessitates participants to be actively engaged, cooperative, and capable of executing specific tasks. However, these demands could pose challenges for individuals with CRCI, potentially leading to frustration or data collection failure. As a more accommodating alternative, rs-fMRI allows us to gather data without placing such cognitive demands on the participant [42]. (2) Task Selection Complexity: Employing tb-fMRI mandates the careful selection of the cognitive task to align precisely with the research objectives and to avoid unintended confounding factors in the results [43]. (3) Comparative Analysis: Utilizing rs-fMRI aligns with the existing research [44,45,46], which has employed rs-fMRI in the field of CRCI and mild cognitive impairment, enabling us to draw comparisons and contextualize our findings. In essence, the adoption of rs-fMRI was a strategic decision that accommodates the unique challenges posed by CRCI patients. 

For resting-state functional connectivity (RSFC) analysis, we conducted a seed-based region of interest (ROI) to ROI approach, which allowed us to delve into the connectivity between the stimulation site and various other regions within the brain. The preprocessed low-frequency fMRI data were parceled into a set of 166 brain regions using Automated Anatomical Labeling (AAL) atlas 3 [47], including salience networks, default mode networks, etc. The RS-fMRI data were preprocessed (including slice-timing and motion corrections, normalization, and the regression of non-neuronal signals) using the CONN toolbox v20b (www.nitrc.org/projects/conn, RRID:SCR_009550, accessed on 20 September 2021) [48]. For the temporal band-pass filtering, the default preprocessing is to remove frequencies below 0.008 Hz and above 0.09 Hz. The fMRI time series was averaged within each brain region. We used Pearson correlation as the metric of association between the time series to estimate functional connectivity between ROIs. The inter-regional correlation coefficient values were transformed into z-values with Fisher’s r-to-z transformation [49]. 

To assess subjective symptomology, the patient was given a brief questionnaire on quality of life based on relevant items from the National Cancer Institute Patient-Reported Outcomes version Of The Common Terminology Criteria For Adverse Events (PRO-CTCAE™) (version 1.0) at 30, 90, and 120 days post treatment. PRO-CTCAE is a tool designed to capture patient-reported side effects and adverse events during cancer clinical trials. While PRO-CTCAE primarily focuses on the assessment of physical symptoms, it can be adapted to evaluate various aspects, such as sleep quality, mood states, and cognitive functions, including memory and attention. Previous oncology studies have employed PRO-CTCAE to specifically address ‘chemobrain’ [50] and PRO-CTCAE has commonly been utilized in phase I trials assessing the cognitive function of cancer patients [51]. Depending on the evaluation items, attributes may include frequency, severity, amount, interference, or presence/absence. Questions were asked in the general format of “In the last 7 days, what was the severity of your (symptom) at their worst?”, followed by “In the last 7 days, how much did (symptom) interfere with your usual or daily activities?” Possible responses for severity were “none, mild, moderate, severe, or very severe” and were “not at all, a little bit, somewhat, quite a bit, very much” for how much interference. 

## 4. Results

### 4.1. Stimulation Feasibility

The original target stimulation intensity of 70% RMT was uncomfortable for the participant during the first session. The TMS coil was reoriented to 55° rotation from the midline and the intensity lowered to 60% of RMT to accommodate discomfort. The intensity was gradually raised over the course of 10 sessions. An intensity of 68.6% RMT was used by the seventh session and 70% RMT by the ninth session. For 10 sessions, the mean intensity of stimulation was 66.0% RMT. With these adjustments, 10 consecutive weekday treatments were successfully completed. Notably, there were no adverse events reported except for transient pain or discomfort at the site of TMS stimulation at the first session.

### 4.2. Neuropsychological Testing

Neuropsychological testing data were acquired before and after treatment (Table 1). 

**Figure 1:**
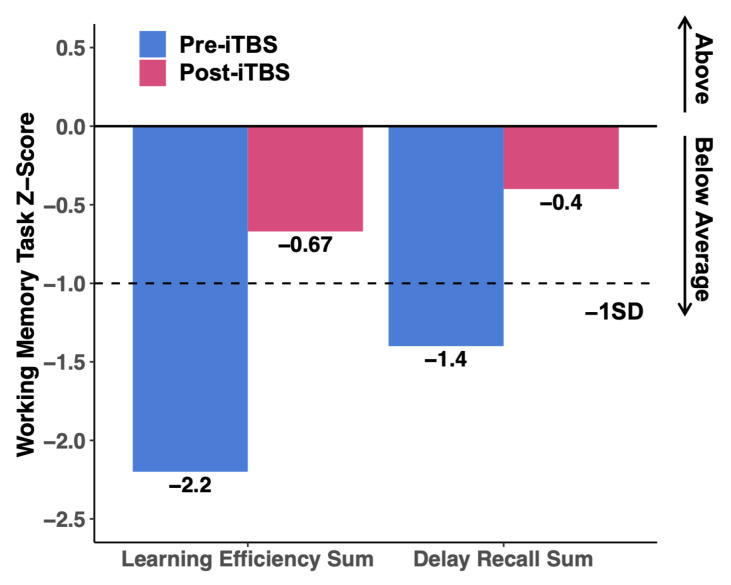
Z-scores of the Rey auditory verbal learning test before and after iTBS.

The participant showed an improvement in memory function (including components of immediate recall, delayed recall, learning, and response inhibition) assessed by the RAVLT. Before iTBS treatment, the participant recalled a total of 49 items on the RAVLT task. After treatment, the score increased to a total of 61 items on the RAVLT. Several scores were derived from the RAVLT, including the Learning Efficiency Sum (LES), which measures the ability to recall words after several repetitions, and the Delayed Recall Sum (DRS), which measures the capacity to recall the repeated words after a 20 min delay [34,35]. The participant’s LES and DRS improved by 1.53 and 1.00 standard deviations (adjusted for age), respectively. Figure 1 illustrates the increase in performance using z-scores, with scores normalized for age. Practice effects in cognitive testing frequently complicate score interpretation across repeated assessments. We addressed this issue by randomly selecting validated alternative forms for Digit Span, Stroop Task, and RAVLT [52]. Notably, the changes in other cognitive tests by z-score all remained within one standard deviation, leading us to assume that practice effects did not significantly impact RAVLT results.

### 4.3. Subjective Reporting

The patient answered a questionnaire on quality of life before and after TMS. She reported “moderate” problems with concentration which interfered with her usual or daily activities “a little bit”. Likewise, she also reported “moderate” problems with memory which interfered with her usual or daily activities “a little bit”. After the treatments with iTBS, she reported no difference in these difficulties with concentration and memory, indicating that the patient-reported attributes remained unchanged. The only symptom that improved after iTBS was the quality of her sleep, which improved from “fairly bad” to “very good”.

### 4.4. Resting-State Functional Connectivity

**Figure 2:**
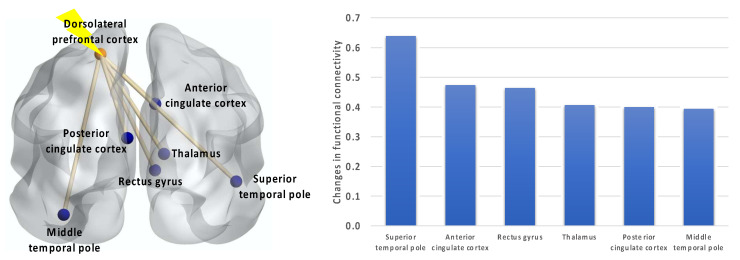
Following iTBS, functional connectivity strength increased between the stimulation site (i.e., the left dorsolateral prefrontal cortex) and the top 6 out of 166 brain regions.

Comparing the fMRI data before and after TMS showed increased resting-state functional connectivity between the stimulation site (i.e., left DLPFC) and several brain regions, such as the right superior temporal pole, right anterior cingulate cortex, right rectus gyrus, right thalamus, left posterior cingulate cortex, and left middle temporal pole (Figure 2).

## 5. Discussion and Conclusions

This first patient in the phase 1 clinical trial testing of TMS for the treatment of “chemo brain” showcased that TMS can be safely administered to individuals with chemo brain, with no significant side effects observed. Noticeably, TMS has the potential to ameliorate impaired cognitive function by altering functional connectivity, providing important lessons for feasibility and insights into the mechanisms of potential benefit. Her pre-treatment mild cognitive impairment objectively normalized after TMS, specifically improving a full standard deviation or more in tests of verbal memory (including components of immediate recall, delayed recall, learning, and response inhibition). These results show the importance of objective testing for evaluating efficacy, since the patient reported no subjective change in her difficulties with memory and concentration. This lack of subjective response may be due to her long-term adaptation to these symptoms, with coping strategies such as memory aids and remaining in the same position at work for many years; thus, these symptoms interfered with her daily life only “a little bit”. Remarkably, after 6 years of chemo brain and remaining in the same position at work due to her inability to concentrate and multi-task, she applied for and received a promotion 5–6 months after her TMS treatments. While impossible to directly attribute her TMS treatments to this momentous life change, it could be more than just coincidence.

Previous studies have reported that cancer patients displaying symptoms of “chemo brain” typically show a relatively distributed pattern of functional connectivity changes, mostly in the frontal and parietal regions of the brain [53]. After TMS, the patient’s functional brain MRI showed an increase in resting state functional connectivity between the stimulated left DLPFC and multiple brain regions involved in memory [54], as well as social and emotional processing [54,55]. In greater detail, the temporal pole (TP), located within Brodmann Area 38 (BA 38), is characterized by complex connections with both the amygdala and the orbital frontal cortex, accordingly rendering it a part of the paralimbic system [55]. The connection between the TP and PFC is facilitated by a significant white matter tract known as the uncinate fascicule. The TP is a versatile brain hub, contributing significantly to an array of cognitive functions, including emotional and social behavior, semantic processing, memory, and the intricate tapestry of human language [54]. The anterior cingulate cortex (ACC, BA 24, 25, 32, and 33) and the rectus gyrus (BA 11 and 12) are components of the neuroanatomical networks associated with DLPFC, where they interact with or exert modulation over emotional regulation processes [56]. Situated within the cingulate cortex, the posterior cingulate cortex (PCC), encompassing BA 23, 29, 30, and 31, serves as a pivotal node within various intrinsic connectivity networks, such as the default mode network, salience network, and sensorimotor networks [57]. PCC is notably susceptible to alterations in connectivity across a spectrum of neuropsychiatric disorders, including depression, Alzheimer’s disease, autism, and many others [57]. A CRCI case report noted a reduction in functional connectivity involving the PCC related networks [58]. It is worth noting that our results showed alterations in connectivity which extended to the thalamus, as well. DLPFC is known to connect reciprocally with the thalamus [59]. The connection between limbic system structures and the anterior nuclei of the thalamus endows the thalamus with a role in various cognitive processes, including learning and episodic memory [60]. Furthermore, the thalamus plays a pivotal role in the regulation of sleep and wakefulness, with inputs received from the PFC contributing to this regulatory function [61]. Our exciting findings have demonstrated that TMS restored the disrupted connectivity of DLPFC. A systematic review [62] has revealed that TBS possesses the capability to reverse functional connectivity, thereby exerting an impact on the functioning of diverse brain regions. These findings align with the hypothesized long-term depression (LTD) and long-term potentiation (LTP)-like plasticity effects. 

In conclusion, these exciting results may explain the changes in memory and quality of life observed in the patient with CRCI. Given this specific predilection for damage to the frontal and parietal regions of the brain and the central role of the frontal–parietal network in working memory, the observed strengthening of the connectivity of the stimulated left DLPFC fits well with the objectively measured improvement in learning and memory performance and with subjective enhancements in the participants’ self-reported sleep quality.

## 6. Future Directions and Limitations

This research is in its infancy and will require much more study to answer critical questions. For example, how long will the benefits of the treatments last? What is the variability amongst individuals for testing and treatment? Will patients with long-term CRCI benefit from chronic treatments akin to those performed for other neurologic diseases? Will TMS be effective in patients with various types of cancer and treatment regimens (chemotherapy, immunotherapy, hormonal therapy, etc.) that contribute to CRCI? It will be interesting to investigate whether patients with a more acute onset of CRCI report a greater subjective response in correlation with objective response. Our participant did report significant improvement in the quality of her sleep, which raises the possibility that TMS’s beneficial effect on sleep may at least partially mediate its potential efficacy. Future research with wearable sensor technology or sleep laboratory studies may therefore be useful. Moreover, the limitations of the study are multifaceted, including but not confined to the following: firstly, its design’s absence of sham control and its non-blinded nature, which thus opens the door to the potential influence of the placebo effect on short-term cognitive enhancement and specific brain regions, such as the DLPFC and the anterior cingulate cortex [63], as delineated in our prior results. Secondly, the limited sample size in this phase 1 clinical trial highlights the necessity for subsequent phase 2 clinical trials with larger sample sizes and a more comprehensive consideration of additional variables. Such measures are quintessential to not only substantiating efficacy, but also for the diligent monitoring of any adverse events.

The impact of this research may extend to other diseases that could benefit from improving the functional pathways involving memory and processing. Given the magnitude and devastation of the COVID-19 pandemic, it is interesting to suggest that our findings may be relevant to the treatment of the lasting neurologic complications from COVID-19. The similarities between long COVID and CRCI may extend beyond the similar “brain fog” described by patients and reach the level of similar structural and functional abnormalities. A study from the UK Biobank analyzed brain scans from patients pre- and post-infection. Compared to non-hospitalized COVID-19 patients, hospitalized COVID-19 patients had a greater loss of gray matter in the amygdala and hippocampal regions, which are involved in the processing of emotional stimuli and memory, respectively, but these results did not achieve statistical significance, possibly since the hospitalized group had only 15 patients [64]. Indeed, a small clinical trial with 12 patients has reported initial promising results for the use of TMS to treat the fatigue and cognitive dysfunction from long COVID-19 [65]. Combining imaging modalities such as MRI with TMS may allow for precision medicine by selecting disease states or individual patients that will more likely respond to TMS and thus streamline the larger clinical trials necessary to provide new hope to patients with chemo brain and other cognitive disorders.

## Figures and Tables

**Table 1:** Neuropsychological data before and after 10 sessions of intermittent theta burst stimulation (iTBS).

Cognitive Domains	Tasks	Pre-iTBS *	Post-iTBS *
Working Memory	Forward Digit Span	7	8
	Backward Digit Span	8	8
Verbal Memory	RAVLT **	49	66
Semantic Memory	Verbal Fluency Task	33	32
Cognitive Flexibility, Response Inhibition	Stroop Color-Word Interference Task	33	36

* All scores are the raw scores (number of items answered correctly). A higher score indicates better performance. ** RAVLT = Rey Auditory Verbal Learning Test.

## Data Availability

Due to the nature of this research, the participant of this study did not agree for their data to be shared publicly, so supporting data are not available.

## Abbreviations

Automated anatomical labeling (AAL); chemotherapy-related cognitive impairment (CRCI); delayed recall sum (DRS); dorsolateral prefrontal cortex (DLPFC); functional MRI (fMRI); gray matter density (GMD); intermittent theta burst stimulation (iTBS); learning efficiency sum (LES); magnetic resonance imaging (MRI); Mayo’s older Americans normative studies (MOANS); parametric estimation via sequential testing (PEST); positron emission tomography (PET); regions of interest (ROIs); repetitive TMS (rTMS); resting-state functional connectivity (RSFC); Rey auditory verbal learning test (RAVLT); transcranial magnetic stimulation (TMS); uniform data set version 3 (UDS3).
